# A Detailed Study of Patent System for Protection of Inventions

**DOI:** 10.4103/0250-474X.45390

**Published:** 2008

**Authors:** G. Krishna Tulasi, B. Subba Rao

**Affiliations:** M. N. R. College of Pharmacy, M. N. R Nagar, Fasalwadi, Sangareddy-502 294, India

**Keywords:** Intellectual property, patents, TRIPs, world trade organization

## Abstract

Creations of brain are called intellect. Since these creations have good commercial value, are called as property. Inventions are intellectual property and can be protected by patents provided the invention is novel, non-obvious, useful and enabled. To have fare trade among member countries, World Trade Organisation proposed TRIPS agreement. India had taken necessary initiation by signing the World Trade Organisation agreement and transformed to global needs. The aim of this article is to enlighten pharmaceutical professionals especially in the field of research and development about planning inventions by thorough review of prior-art, which saves time and money. A thorough understanding is made possible by providing details of origin; present governing bodies, their role along with the Act that is safeguarding the patent system.

The world patent has been coined from a Latin word patent-em meaning open. A patent is a document issued by government to the inventor granting permission to exclusively make, use and sell on disclosure of the invention for a definite period of time. Unlike patents, monopoly existed where inventions were not disclosed and exclusively sold.

## ORIGIN OF PATENT SYSTEM

In 1300s, the first person who found resources in the Alps dictated property rights for mining, timber and water. As competition progressed special privileges were granted for useful creation. In 1409, first patent was granted to a German for the construction of a model mill. A monopoly by the British was not granted to sell playing cards due to obviousness. The first English patent was granted for a period of 20 year to John of Utynam on making stained glass. Meanwhile, the French advanced the system by registration and examination. In the United States, a patent was granted for a grain elevator ‘hopper boy’ to Oliver Evans.

In the Indian context, in 1856, the Act VI^1^ on protection of inventions based on the British Patent Law of 1852 was established. During this period certain privileges were granted to inventors of new manufacturers for a period of 14 year. In 1859, the act was modified as Act XV in which making, selling, using of inventions in India and authorizing others to do so for 14 year from the date of filing the specification. In 1872, the act was re-named as The Patents and Design Protection Act, in 1883 as The Protection of Inventions Act, in 1888 consolidated as The Inventions and Designs Act and in 1911 as The Indian Patents and Designs Act.

## REASONS FOR THE ORIGIN OF PARIS CONVENTION, BIRPI^2^

Initially, inventions were kept secret so that it is well protected. As technology developed periodically, as a matter of national prestige the inventions were exhibited. At the Paris exhibition in 1867, Germany received the first genuine recognition as an industrial nation. During the 1873 Vienna exhibition, it was the Americans who refused to participate. The reason was that the Americans need intellectual protection of their creations from German nations so that the ideas are well protected. This led to the origin of Paris convention in 1883. This international treaty helped people of one country to protect their creations in another country, provided the other country is also a member of the convention. The main advantage is that the inventor has the right of priority of his invention. This in turn was the origin of the protection of industrial property in different countries. In 1893, in order to carry out the administrative tasks, an international organization called United International Bureaux for the Protection of Intellectual Property (BIRPI) was established in Berne, Switzerland.

## REASONS FOR FORMATION OF GATT AND WIPO^2^

After World War II, economy in many European and Asian countries was shattered. After the UNO was born, three bodies were born in 1947, i.e., World Bank, International Monetary Fund (IMF) and International Trade Organization (ITO). It was the US senate, which blocked the ITO. The objective of these organizations was to revive the economy especially in developing countries. It was in the same year India signed General Agreement on Tariffs and Trade (GATT). The agreement was designed to provide international free trade within member states by regulating and reducing tariffs on traded goods. The main objective was to encourage trade. On January 1, 1948, 23 contracting states including India ratified GATT. Mean while, with the increasing awareness of the intellectual property, in 1960, in order to bring closer to United Nations and other international organizations, BIRPI was shifted from Berne to Geneva. In 1967, in order to modernize and for better administration of the unions with respect to protection of the intellectual property and artistic works, while fully respecting the independence of each of the union, World Intellectual Property Organization (WIPO)^3^ replaced the BIRPI.

## ROLE OF GATT

It was under GATT, the biggest advancement in international trade liberalization have come in to existence through multilateral trade negotiations. The role^4^ of GATT is to provide a stable and predictable international trade system. Secondly, it acts as a mediator in settling the disputes between countries regarding trade. Thirdly, it holds frequent negotiations, encourages reductions in tariffs so that expansion in world trade becomes possible. The objective of India signing the GATT agreement is to export indigenous products and in turn purchase oil, industrial raw materials, machines, new technology and other things that are domestically needed. During 1950s and 1960s, continuous reductions of tariffs led to high rate of world trade growth. Thus in the GATT era, trade liberalization helped in trade growth consistently instead production growth.

## ROLE OF WIPO

The role^2^,^5^–^7^ of WIPO is to promote international cooperation with respect to creation, dissemination, use and protection of works of the human mind for economic, social, cultural progress of all mankind. It enhances a worldwide balance of the creation i.e., by protecting moral, material interests of the creators and providing access to the socio-economic and cultural benefits to others. WIPO promotes protection of intellectual property and bring out cooperation among the union. In addition to these, WIPO sets norms, standards and execute legal technical assistance, registration activities for intellectual property protection to member countries. It is the WIPO; which is responsible for the formation of Patent Co-operation Treaty (PCT).

## REASONS FOR FORMATION OF PCT

Basically, under the traditional patent system, the inventor has to file applications in each and every country where he wishes to possess a patent. The Paris Convention provided a great opportunity in claiming the priority date of an earlier application for the subsequent filings in foreign countries if the parent and foreign countries are members of the convention. The advantage with the Paris Convention is that, the inventor after filing a patent in his/her own country can decide within a period of 12 months whether to file patent applications among Paris Convention countries. This in turn means that the inventor if wishes to file patent application in foreign countries; within a period of 12 months he/she has to make all the necessary arrangements of language translations, filing of patent applications in all the countries, bare fees of patent offices, attorney's. To overcome the problem of duplication, BIRPI/WIPO came out with a new treaty called Patent Cooperation Treaty in 1970. PCT became an international cooperation treaty with respect to filing, searching, and examination of patent applications and dissemination of the technical information contained in it.

## ROLE AND OBJECTIVE OF PCT

Patent Cooperation Treaty brings out several benefits for the users of patent system i.e., brings one application filing with one single language which in turn is valid in PCT member countries, provides single time examination of the patent instead each member country, provides international search rather than each country search so that prior art can be easily judged in order to get a patent, provides international publication of international publications with related international search reports, bring down one single communication to all designated offices, provides any person from the member country to file single opposition regarding the patentability of the invention, provides uniform procedure and economical benefit to the inventor in all mentioned aspects. In addition to these the main objective of PCT is to facilitate and accelerate access by industries and other sectors to technical information relating to inventions and to assist developing countries in gaining access to technology.

## REASONS FOR THE PATENTS ACT, 1970

Medicines required for human cure were not easily obtained to the public in India. The major source of these medicines was from foreign countries. The lack of indigenous medicines and their huge demand led to very high prices. It was the external law that influenced the local law. Drug prices in India were amongst the highest in the world. In 1957, the Indian Government appointed Justice Rajagopala Ayyangar committee to revise the patent law to comply with the then, industrial needs. The report suggested for a process patenting so that the medicines reach even the poor sections of the society. The government vested the Patents Act in 1970. This revolutionized the economic system in India by providing the medicine at a low price.

## UNDERSTANDING THE PATENTS ACT, 1970^8^

One should review the Patents Act, 1970 and understand how different aspects of intellectual property were considered at that moment. Section 3, 5 of the Patents Act, 1970 mentions inventions that are frivolous, obvious, exploiting commercially to public, immoral, prejudice to human, animal, plant life or health or to the environment, scientific principles, abstract theories, identified to possess new use for a known substance, known process, known machine or known apparatus, aggregation of the properties by admixture and process for production of such substances, arrangement or re-arrangement or duplication of known devices, methods of agriculture, horticulture, processes for the medicinal, surgical, curative, prophylactic or other treatment of human beings, animals to render them free of disease or to increase their economic value or that of their products, a mathematical or business method or a computer programme *per se* or algorithms, literary, dramatic, musical, artistic work, cinematographic works, television productions, rule or method of performing mental act, method of playing game, presentation of information, topography of integrated circuits, aggregation or duplication of known properties of a traditional knowledge, atomic energy, claiming substance intended for use, or capable of being used, as food or as medicine or drug, or substances prepared or produced by chemical processes are not patentable.

## HOW INDIA MADE USE OF THE PATENTS ACT, 1970

With the provisions made in the Patents Act, 1970 the citizens of India got an opportunity to develop processes. This led to a huge benefit in economic growth of India. Bulk drug manufacturers made use of this opportunity in manufacturing drugs in bulk and selling at a cheaper price.

## GATT TUNING TO WTO

In 1970s despite, GATT's success in trade growth through tariff reductions, global competition led a series of economic recessions leading to high rate unemployment, factory closures. To overcome this, governments were driven to devise other forms of protection i.e., bi-lateral market sharing agreements within competitors and embark subsidies to maintain holds on agricultural trade. In addition to this, advancements in science, individual needs, world trade became complex. Trade services were found promising globalization of world economy, but rules not covered in GATT. These changes undermined the credibility and effectiveness of GATT. Together, these and other factors influenced among GATT members and concluded to vest multilateral system. This led to Uruguay round of negotiations; the last and largest round of GATT.

## URUGUAY ROUND^2^,^9^

Having found some set backs in the rules of GATT, the members came together to negotiate issues regarding international trade liberalization, improve the rules. Starting in 1986, the negotiations ended in 1993. Not only the inclusion of the traditional areas of trade in goods, the rules relating, trade in services, trade-related intellectual property rights (TRIPS) in the negotiations, but also the approval of farm trade by services, market access, anti-dumping rules and the proposed creation of new institution by all member countries dragged attention on Uruguay round. It was on April 15, 1994 at Marrakesh, Morocco, the ministries of 125 governments signed the agreement.

## WTO, TRIPS ORIGIN^2^

The agreement signed at Marrakesh, Morocco led to replacement of GATT by the World Trade Organization (WTO), on January 1, 1995. Upon signing the agreement all the countries became WTO members. Under the annex 1C of WTO agreement an agreement regarding Trade Related Aspects of Intellectual property has been included.

## ROLE OF WTO^2^

The World Trade Organization mainly deals with agriculture, textiles, clothing, banking, telecommunications, government purchases, industrial standards and products safety, food sanitation regulations and intellectual property. The principle^2^,^4^ is foundation of multilateral trade system by treating foreigners and locals equally, bringing free through negotiations, predicting through binding and transparency, promoting fair competition, encouraging development and economic reforms.

To improve welfare of the people of the member countries WTO bring benefits like peace, solving disputes among countries, free trade that in turn reduce cost of living, choice of products, quality, economic growth and good government. Thus in turn WTO improves welfare of people of the member countries.

## HOW WTO DIFFERENT FROM GATT

Despite GATT's commitments are being provisional; the WTO is being complete and permanent. GATT rules were applied to trade in merchandise goods where as the WTO rules were applied to trade in services, trade-related aspects in intellectual property additionally. In case of dispute settlements, the WTO system of dispute settlement is faster, automatic and less susceptible to blockage when compared with GATT. In addition to these, the ‘GATT 1947’ will continue to exist until the end 1995, meanwhile ascending the members to WTO. One should keep in mind that the amended version of GATT 1947 i.e., “GATT 1994” exits as an integral part of the WTO agreement. In 1974, WIPO became a specialized agency of the United Nations system of organizations to administer the intellectual property matters recognized by the member states of the UN.

## REASON BEHIND ORIGIN, ROLE OF TRIPS^2^

The wide variation in the standards and protection of intellectual property, lack of multilateral frame of principles, rules and disciplines dealing with international trade led to tensions in international economic relations. In order to solve these tensions, an agreement i.e., Trade Related Aspects of Intellectual Property Rights was framed addressing basic GATT principles and those of international intellectual property agreements was brought under WTO. Thus a provision of adequate intellectual property rights, effective enforcement measures of those rights, multilateral dispute settlements and transitional arrangements were framed.

## WHEN INDIA REALIZED MORE ABOUT INTELLECTUAL PROPERTY

### Issue of Turmeric patent^10^,^11^:

Turmeric is a tropical herb grown in east India. Turmeric powder is widely used in India as a medicine, a food ingredient and a dye to name a few of its uses. United States awarded patent on turmeric to University of Mississippi medical center in 1995 for wound healing property. An exclusive right has been granted to sell and distribute. Two years later, India's Council of Scientific and Industrial Research challenged the university regarding the novelty of the discovery. The USPTO cancelled the patent due to lack of novelty.

### Issue of Basmati rice patent^12^:

Basmati rice is a famous cereal of India and is well known for its fragrant taste. It is this rice, which is grown in Basmati region of India possessing its fragrant taste. This rice was well developed by Indian farmers over hundreds of years, but, a Texan company obtained a patent for a cross breed with American long-grain rice. The US granted a patent to the company on the basis of aroma, elongation of the grain on cooking and chalkiness and since then the company can charge the farmers to grow the rice and not allow planting the seeds for the following year's crop. This made India to realize and file a petition with scientific evidence in the United States Patents and Trademarks Office saying that most varieties of Basmati possess these qualities. The USPTO accepted the petition.

### Issue of Neem patent^13^,^14^:

W.R. Grace and the Department of Agriculture, USA has first filed a patent in European Patent Office. The patent is a method of controlling fungi on plants comprising of contacting the fungi with a neem oil formulation. A legal opposition has been filed by India against the grant of the patent. The opponents' submitted evidence that hydrophobic extracts of neem seeds were known and used for centuries in India, both in curing dermatological diseases in humans and in protecting agricultural plants form fungal infections. Since then, traditional Indian knowledge is in public. The EPO identified the lack of novelty, inventive step and possibly form a relevant prior art and revoked the patent. In addition to this patent, several other patents dealing with neem are pending.

## DRAWBACKS IN PATENTS ACT, 1970

Indians have the attitude of sharing the knowledge to others without protecting it, which is in turn, a major draw back. Thus there was no mention in the Patents Act, 1970 for the protection of products such as Darjeeling tea and Basmati rice, which are famous for their superior quality from that geographical location. In addition to these, since ancient times some plant parts of turmeric, neem were well used as medicine, but there is no mention in the Patents Act, 1970 for the protection of the ancient knowledge which is being used since generations.

## ASPECTS INDIA MUST COMPLY WITH REFERENCE TO TRIPS

The dispute in case of turmeric, neem and basmati brought awareness and the debate made to realize the need for strong intellectual property laws. The conclusion of the Uruguay round in 1994 paved the way for more change in this area of law and India joined the World Trade Organization and became obligated to comply with the Trade Related Aspects of Intellectual Property Rights (TRIPS). TRIPS provided a three-stage time frame by introduction of mailbox facility, exclusive marketing rights and product patent for India to comply with the obligation.

## PATENTS (FIRST AMENDMENT) ACT, 1999, DT. 26-3-1999 W.E.F 1-1-1995^15^–^17^

Under the amendment of this act from January 1, 1995, a product patent application can be filled provided the product is used as medicine or drug, except intermediate. Since, India is having a ten-year transition period, the application for the product patent cannot be processed until end of 2004. A major provision is made under section 24 A, B of chapter IV A of the Patents Act, 1970 for a grant of Exclusive Marketing Right provided certain conditions are fulfilled for a period of 5 year.

## MAILBOX FACILITY^15^,^16^

India has to amend its Patent Act, 1970 for availing patent protection for products. From January 1, 1995 till January 1, 2005, an application can be filed for a product patent under the provision of mailbox and after January 1, 2005 the applications will get examined and those that comply are granted product patents.

## DOHA DECLARATION^18^

The Doha ministerial conference in November 2001 was considered as a success. The new round is a challenge, opportunity for developing countries to get more favorable concessions under the emerging regime. One of the important aspects was the implementation of obligations under TRIPS by member countries. The Doha declaration also solved the issue of non-affordability of patented products especially to half of the world's population. The members agreed that each member has the right to grant compulsory licenses, with the freedom to determine the grounds upon which such licenses are granted and can determine what constitutes a “National Emergency” or other circumstances of “Extreme Urgency”. The provision made speedy issue of compulsory license where needed. Another point of concern among pharmaceutical companies is the interpretation in some countries that the diseases indicated in the declaration, namely HIV/AIDS, Malaria and TB are only illustrative and the country concerned would be free to consider any disease of grave public health concern to be added. However, several countries raise concern regarding the abuse of the provisions.

## PATENTS (SECOND AMENDMENT) ACT, 2002,DT.20-5-2003 W.E.F. 20-5-2003^15^,^16^

To cross the barriers of TRIPS other than EMR, India introduced second amendment bill and several changes were made in the Act. The following are the key changes made.

### Patentable inventions:

Section 2 of the Patents Act, 1970, define invention as a new product or process involving an inventive step and capable of industrial application. With the proposal of introduction of product patents, the definition is broadened by introduction of the words ‘non-obvious’ and ‘useful’ synonymous to inventive step and capable of industrial application, respectively.

### Not inventions:

A provision is made in Section 3 of the Patents Act indicating that plants and animals in whole or any part thereof other than micro-organisms but including seeds, varieties and species and essentially biological processes for production or propagation of plants and animals as not inventions.

### Exclusions from patentability:

Section 3 of the Patents Act, 1970 clears that are not inventions. The new definition excludes, in sub-section 3, inventions whose ‘primary or intended use or commercial exploitation’ is contrary to law and morality. In addition to this, the bill also excludes medical, surgical, curative, prophylactic, diagnostic, therapeutic treatments for humans, plant and animals. India also excludes the patenting of computer software, business method patents specifically and biotech patents.

### Term of date of patent:

Earlier the patent protection term was 14 years from the date of filing the complete specification of the patent. However, the proposed bill extended the term to 20 years.

### Application requirements:

Section 10 of the Patents Act, 1970 mainly clears about the contents of the specifications in a patent. The section mainly tells that a patent must include with title, description of the invention, drawings, model or sample of anything illustrating the invention, description of the operation, use, method of performance of the invention, the best method of performance, ending with a claim defining the scope of the invention. The amendment includes additionally an abstract of the invention, which must be included in to the patent.

### Compulsory licensing:

The chapter XVI of the Patents Act, 1970 provides compulsory licensing as a necessary safeguard for protecting public interest. After 3 year of a sealed patent, any interested party can request for a compulsory licensing provided the invention is not reasonably available to the public. Central government has the right to make an application requesting the controller to endorse a patent with the ‘license of right’. The amendment removed the provision of the central government. In addition to this necessary amendments were done with reference to requirement of the public are not satisfied, if the invention is not manufactured in India or the patentee refuses to grant a license by removing a presumption that requirements of the public are not satisfied based on local manufacture. The amendment also made a provision to the controller for granting compulsory license under conditions of national emergency. A provision has also been made enabling the third party to seek for compulsory license even the invention is not manufactured in India. The amendment also provides the revocation of the compulsory license by the controller himself if the circumstances that gave raise to it cease to exist.

### Right to import and parallel imports:

The second amendment act mentions a right to import patented products from an authorized license holder indicating that the provision is not an act of infringement. Thus the Patents Act, 1970 that did not vest on the patentee or a license holder the right to importation a patented product is dissolved. This made an accessibility of products in all ranges of cost in India. However, importing a patented product from the patentee, valid license holder will amount to infringement under TRIPS. The doctrine of exhaustion specifies that the patent holder does not have any control over a buyer or a licensee once the product has been placed in the market. The implied license suggests that a buyer can remanufacture the goods and import them into the same market for a lesser cost thus restricting spurious parallel imports into the country and balance the effect of taking away the need for local production and will also be in line with TRIPS.

### Bolar provisions:

A provision has been made under the Patents Act, 1970 for the submission of data relating to bio-equivalence of drugs before the expiration of the patents. The stock piling before the expiry of the term of the patent is prohibited.

### Burden of proof:

A provision has been made under the Patents Act, 1970 as burden of proof in case of suits concerning infringement of a patent in which the subject matter is a process for obtaining a new product. Under such conditions the court may ask the defendant to prove that the process used by him to obtain the product, is identical to the product of the patented process is different from the patented process.

## THE PATENTS (AMENDMENT) ORDINANCE, 2004^19^

As part of the three-stage frame to comply with TRIPS, India passed the Patents (Amendment) Ordinance, 2004. In section 2 of the Patents Act, 1970 definition and interpretation of Budapest Treaty was included. It means the Budapest Treaty on International recognition of the deposition of microorganisms for the purposes of patent procedure done at Budapest on April 28, 1977, as amended and modified from time to time. Section 3 of the Patents Act, 1970 relating to inventions where only methods or processes of manufacture patentable are omitted. The Act gave provisions for patenting a process as well as a product.

In section 7 of the Patents Act, 1970 the filing date of an application referred to in sub-section (1A) shall be considered as the international filing date accorded under the “Patent Cooperation Treaty”. The sections 22, 23 and 24 dealing with acceptance, advertisement and effect of acceptance of complete specification are omitted. The sections 24A, 24B, 24C, 24D, 24E and 24F of the Patents Act, 1970 dealing with mainly Exclusive Marketing Rights are omitted indicating that a provision being made during the transition period. A new section 92 A, was introduced for grant of compulsory license for manufacture or export to any country, which has insufficient, or no manufacturing facility provided an application is made by the convention country.

## CONCLUSION

It is not until recently every common man is aware of intellectual property rights. A thorough clear understanding of intellectual property rights is necessary where industrial property and copyright is well protected that in turn raise the economy of the country. The Government of India provided the entire infrastructure. Special provisions were made in protecting software, traditional knowledge, plant varieties, and geographical indications. A thorough understanding regarding the intellectual property rights helps in quick and easier identification, planning, execution and protection of creativity.
